# Effects of Varying Antagonist Exercise Volume in Upper-Body Supersets on Mechanical, Metabolic, and Perceptual Responses in Resistance-Trained Men

**DOI:** 10.3390/jfmk10040419

**Published:** 2025-10-23

**Authors:** Gonzalo Márquez, Etham Coutado-Sánchez, Adrián Villaraviz-Ferro, Daniel Marcos-Frutos, Amador García-Ramos, David Colomer-Poveda

**Affiliations:** 1 Department of Physical Education and Sport, Faculty of Sports Sciences and Physical Education, University of A Coruna, 15179 A Coruña, Spain; etham.coutado@udc.es (E.C.-S.); adrian.villaraviz@udc.es (A.V.-F.); 2 Department of Physical Education and Sport, Faculty of Sport Sciences, University of Granada, 18011 Granada, Spainamagr@ugr.es (A.G.-R.); 3 Department of Sports Sciences and Physical Conditioning, Universidad Católica de la Santísima Concepción, Concepción 4030000, Chile; 4 Centre for Sport Studies, Rey Juan Carlos University, 28943 Madrid, Spain; david.colomerp@urjc.es

**Keywords:** velocity-based training, strength training, neuromuscular fatigue, antagonist exercises, training efficiency, performance

## Abstract

**Objectives**: This study aimed to analyze the effects of varying antagonist volume in upper-body supersets on mechanical (lifting velocity), metabolic (blood lactate), and perceptual (perceived exertion) variables. **Methods**: A randomized crossover study was conducted in which 14 resistance-trained men performed three strength training conditions. In the control condition (CTR), participants performed four sets of bench press with 8 repetitions at their 12-repetition maximum load, whereas in the experimental conditions, a prone bench pull was performed immediately after the bench press using 33% (SS1) or 66% (SS2) of the individual’s maximum possible repetitions. Lifting velocity, lactate concentration, and perceived exertion were measured. Repeated-measures ANOVA or Friedman test was applied to compare conditions, with Bonferroni-corrected post hoc tests and effect sizes reported. **Results**: Despite a progressive decrease in mean set velocity (*p* < 0.001) and fastest set velocity across sets (*p* = 0.014) in the agonist exercise (i.e., bench press), these variables did not significantly differ between conditions. The only difference observed was a lower mean set velocity during the prone bench pull in the SS2 condition compared to the SS1 condition (*p* = 0.011). Perceived exertion also increased across sets (*p* < 0.001), with no differences between protocols. Blood lactate concentration, measured before the final set, was significantly higher in SS2 compared to CTR (*p* = 0.003) and SS1 (*p* < 0.001), indicating a greater metabolic load during training. **Conclusions**: Agonist–antagonist supersets allow for reduced training time without negatively impacting acute mechanical performance in the agonist exercise. Low-fatigue configurations (SS1) in the secondary exercise do not significantly increase lactate levels, while moderate-fatigue configurations (SS2) in the secondary exercise increase metabolic load.

## 1. Introduction

Strength training is an essential tool in both athletic and general health contexts, being recognized for its capacity to improve maximal strength, power, muscular endurance, and various physiological and perceptual markers [1,2,3,4]. In both high-performance environments and the general population, training sessions must be structured to consider not only the effectiveness of the stimuli but also time efficiency. The traditional set configuration, characterized by long rest periods between exercises and sets, remains the most commonly used method in resistance training [1,5]. However, its extended duration may hinder adherence and limit the practical applicability of training programs, especially when time availability is a significant constraint [6].

Supersets (SSs) have emerged as a time-efficient alternative to traditional strength training methods. They involve the consecutive performance of two exercises with minimal or no rest between them [7]. This approach allows for a significant reduction in total session duration without necessarily compromising training volume or kinematic outputs. Among the different types of SS, agonist–antagonist structures (e.g., bench press and prone bench pull) have shown particular promise by allowing active recovery between opposing muscle groups, thereby maintaining mechanical performance while increasing training efficiency [8,9].

A recent systematic review and meta-analysis analyzed the available evidence comparing traditional set structures and SS in terms of both acute and chronic training outcomes [10]. The authors concluded that SSs enable the maintenance of similar repetition volumes and loads compared to traditional structures (SMD = −0.03 and 0.05, respectively), while significantly reducing session duration (SMD = 1.74) and increasing training efficiency. However, SS structures promoted higher lactate concentrations ([La]) (SMD = 0.94 and 1.13 during and post-exercise, respectively), higher energy cost (SMD = 1.93), and increased ratings of perceived exertion (RPE) (SMD = 0.77), suggesting a greater internal load.

The agonist–antagonist SS can increase the total number of repetitions (SMD = 0.68) without compromising total load, likely due to neuromuscular facilitation effects derived from antagonist pre-activation [11]. Despite these advantages, there is limited evidence on the specific impact that different fatigue levels in the antagonist exercise may have on mechanical performance in the primary exercise (the one performed first, prioritized according to training goals), particularly when analyzed from the perspective of lifting velocity, a key marker in velocity-based training programs [12,13,14,15]. Despite advancements, gaps persist in the literature. Specifically, the impact of varying degrees of fatigue in the antagonist exercise on mechanical (e.g., lifting velocity), physiological (e.g., [La]), and perceptual (e.g., RPE) markers has not been systematically studied. This information is key to optimizing SS design, particularly in training contexts aiming for efficiency without compromising performance.

Therefore, the present study aimed to determine the effect of manipulating the number of repetitions performed in the antagonist exercise (prone bench pull [PBP]) on lifting velocity in the paired agonist exercise (bench press [BP]), as well as on blood lactate concentration ([La]) and ratings of perceived exertion (RPE) across the entire training session. Participants completed either 0% (CTR condition), 33% (SS1 condition), or 66% (SS2 condition) of the maximum possible repetitions in the antagonist exercise, potentially inducing different levels of fatigue. We hypothesized that (i) lifting velocity in the BP would decrease progressively across conditions (CRT > SS1 > SS2) and (ii) [La] and RPE would increase progressively across conditions (SS2 > SS1 > CTR), with the largest effect sizes expected between the CRT and SS2 conditions for all variables.

## 2. Materials and Methods

### 2.1. Participants

Fourteen resistance-trained men (age: 21.7 ± 3.1 years; body mass: 82.50 ± 5.56 kg; height: 1.80 ± 0.07 m; training experience: 4.9 ± 2.7 years) voluntarily participated in the study. At the time of the study, all participants were training at least three times per week, were non-smokers, had no neurological, metabolic, and/or cardiovascular disorders, and had no injuries in the previous six months that could affect their ability to perform the experimental sessions. All participants successfully completed the Physical Activity Readiness Questionnaire (PAR-Q) and provided written informed consent prior to participation. They were instructed to avoid strenuous physical activity in the 24 h prior to evaluation and to refrain from caffeine and analgesic intake in the 12 h beforehand. All participants reported having prior experience with superset configurations, as this is a common practice in resistance training settings.

### 2.2. Experimental Design

A randomized crossover design was used, consisting of four sessions over two weeks: one familiarization session and three experimental conditions: CTR, SS1 and SS2. The order of the experimental conditions was randomized and counterbalanced to mitigate order and learning effects. A recovery interval of 3–7 days was maintained between sessions. All sessions were conducted at the same time of day (±1 h) to minimize circadian variation effects.

### 2.3. Procedures

#### 2.3.1. Preliminary Session

Before starting the experimental conditions, all participants underwent a familiarization session with the procedures, exercises, and equipment used in the study. During this session, the BP and PBP techniques were standardized, and subjects were instructed on the importance of performing the concentric phase at maximum velocity. They were also familiarized with the OMNI-RES perceived exertion scale, following recommendations by Robertson et al. [16]. In this same session, both 1RM and 12RM loads were determined as described below.

**Figure 1:**
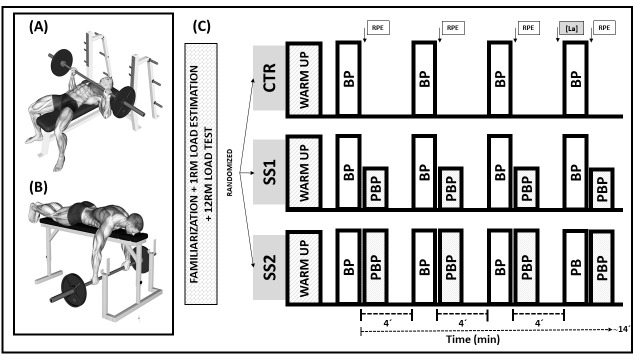
(**A**–**C**) Schematic representation of the study design and session structure. BP: bench press; PBP: prone bench pull; RPE: Rating of Perceived Exertion; [La]: blood lactate concentration.

A progressive loading test was conducted to estimate 1RM in both BP and PBP exercises using a linear encoder (Chronojump-Boscosystem^®^, Barcelona, Spain), following a previously validated and reliable protocol [17]. In BP (Figure 1A), the subject started with elbows extended, lowered the bar in a controlled manner to the chest, and executed the concentric phase at maximal velocity. Grip width was kept constant across sessions. In PBP (Figure 1B), participants lay prone with their chin supported on the edge of a high bench. The pulling phase began with elbows fully extended while the bar was held with a shoulder-width grip. They were instructed to pull with maximal effort until the bar contacted the lower part of the bench (8 cm from the participant’s chest), then lower it back to the initial position; the legs were anchored by one of the evaluators. The concentric phase was always performed at maximal voluntary velocity. A brief pause (~1 s) was introduced between eccentric and concentric phases to minimize bounce effects and allow for more reproducible measurements. Only concentric actions (pressing in BP and pulling in PBP) were analyzed. 1RM was estimated using a velocity threshold of 0.17 m·s^−1^ for BP [18] and 0.47 m·s^−1^ for PBP [19]. Mean absolute 1RM loads were 106.4 ± 21.0 kg for BP and 107.3 ± 15.0 kg for PBP.

Ten minutes after the progressive loading test for 1 RM estimation in both exercises, a 12-repetition maximum (12RM) test was conducted for BP and PBP (10 min apart) to determine training loads. Participants performed a set at 70% 1RM to failure. If they exceeded 12 reps, the load was increased (2.5–5 kg); if not, it was reduced. The test was repeated with 5–7 min rests until the exact 12RM load was found. Mean absolute 12RM loads obtained were 75.4 ± 15.0 kg for BP and 75.7 ± 11.7 kg for PBP.

#### 2.3.2. Experimental Sessions

Before each experimental session, and prior to warm-up, participants completed a wellness questionnaire rating their sleep quality (1 = Insomnia, 5 = Acceptable, 9 = Very restorative), fatigue (1 = Very rested, 5 = Acceptable, 9 = Very fatigued), muscle soreness (1 = I feel great, 5 = Acceptable, 9 = Intense pain), and stress (1 = Very relaxed, 5 = Acceptable, 9 = Very stressed) using a 9-point scale [20].

Following a standardized warm-up, participants performed four sets of eight repetitions at maximal intended velocity with a 12RM load during the BP exercise. In the CTR condition, four traditional BP sets were completed with four minutes of rest between them. In SS1 and SS2 conditions, the PBP exercise was executed using the 12RM load immediately after each BP set (≤10 s). Participants performed 4 repetitions in the SS1 condition (33% of the maximum possible repetitions) and 8 repetitions in the SS2 condition (66% of the maximum possible repetitions). As in CTR, the total time between BP sets was four minutes. The experimental session structure is schematically represented in Figure 1C.

#### 2.3.3. Assessment of Mechanical, Metabolic and Perceptual Variables

Mean concentric velocity of each BP and PBP repetition was recorded using a linear encoder (Chronojump-Boscosystem^®^, Barcelona, Spain), fixed vertically to the bar. Participants were instructed to perform each concentric phase as fast as possible with proper technique, while being supervised and continuously encouraged by the researchers. The eccentric phase was performed in a controlled ~2 s tempo. Statistical analysis used the mean set velocity (MSV = average of all repetitions) and fastest set velocity (V_fastest_ = highest velocity of the set). After each BP set, participants rated their RPE using the OMNI-RES scale, which ranges from 0 (“Extremely easy”) to 10 (“Extremely hard”) [16]. Finally, [La] was assessed via capillary blood sampling from the right earlobe 30 s before the final BP set of each protocol using a LactatePro 2 portable analyzer (ARKRAY Factory Inc., Shiga, Japan), following standardized cleaning, puncture, and sampling procedures [21]. The rationale for collecting blood samples 30 s before the final set was based on methodological and physiological considerations. First, because our main objective was to analyze the influence of antagonist exercise volume on subsequent agonist performance, it was essential that the lactate measurement represented the metabolic status immediately prior to the final agonist set rather than a post-exercise accumulation. Second, although peak lactate concentrations are generally observed between 3 and 8 min after exhaustive exercise [22], in our study, the sampling point corresponded to approximately 3.5 min after the completion of the third set—consistent with the time frame of maximal lactate appearance. Most importantly, lactate was always measured at the same time point across all conditions (CTR, SS1, SS2) and participants, ensuring valid and reliable comparisons of metabolic load.

### 2.4. Statistical Analysis

Normality was assessed using the Shapiro–Wilk test. Only V_fastest_ in PBP showed a normal distribution. After logarithmic transformation, all variables met the normality assumption except RPE and wellness measures (sleep quality, fatigue, muscular pain, stress), for which non-parametric tests were applied.

Inter-session reliability of MSV and V_fastest_ from the first BP set in all three experimental conditions (CTR, SS1, SS2) was evaluated using the coefficient of variation (CV = SD/Mean × 100) and the intraclass correlation coefficient (ICC [1,3], mixed-effects model; 95% confidence intervals). ICC interpretation followed standard thresholds: <0.5 (low), 0.5–0.75 (moderate), 0.75–0.90 (good), >0.90 (excellent).

The Friedman test was used to compare wellness variables (CTR vs. SS1 vs. SS2). When significant (X^2^), post hoc comparisons used Conover’s test. For MSV and V_fastest_ in BP, a two-way repeated measures ANOVA (RM-ANOVA) was used with CONDITION (CTR, SS1, SS2) and SET (SET1–SET4) as factors. For PBP mean and fastest set velocity, a two-way RM-ANOVA with CONDITION (SS1, SS2) and SET (SET1–SET4) was used. For RPE, a non-parametric ANOVA-type test (F1-LD-F1) was applied with CONDITION and SET as factors, followed by Wilcoxon signed-rank post hoc tests. For all repeated measures post hoc analyses, Bonferroni correction was applied to adjust for multiple comparisons.

Effect sizes are presented as partial eta squared (ηp^2^: small = 0.01; medium = 0.06; large = 0.14). Statistical significance was set at *p* < 0.05. Data are reported as mean ± standard deviation in text and tables. Analyses were performed using JASP (v0.19.3) and the R package nparLD (v4.4.1).

## 3. Results

### 3.1. Baseline Wellness

No significant differences were observed between conditions in sleep quality, fatigue, muscle soreness, or stress, indicating comparable initial conditions across sessions (see Table 1).

### 3.2. Reliability of BP Velocity Measures

MSV and V_fastest_ reliability was good-to-excellent with ICC values of 0.932 (95% CI = 0.844–0.976) and 0.870 (95% CI = 0.716–0.952), respectively. CVs were 8.4% for MSV and 7.1% for V_fastest_.

### 3.3. Mechanical Performance During Agonist Exercise (BP)

BP MSV showed a significant SET effect (*p* < 0.001, ηp^2^ = 0.402), indicating progressive velocity decline across sets (Table 2). No significant CONDITION (*p* = 0.422, ηp^2^ = 0.064) or CONDITION × SET interaction effects (*p* = 0.629, ηp^2^ = 0.053) were observed. Post hoc tests showed MSV reductions between SET1 and SET3 (mean difference: 0.047; 95% CI = 0.002–0.092; *p* = 0.038), and SET1 and SET4 (mean difference: 0.068; 95% CI = 0.012–0.123; *p* = 0.014).

BP V_fastest_ also showed a SET effect (*p* = 0.014, ηp^2^ = 0.236), but post hoc comparisons, however, did not reach statistical significance (all *p* > 0.05). Furthermore, no significant CONDITION (*p* = 0.272, ηp^2^ = 0.095) or interaction effects (*p* = 0.831, ηp^2^ = 0.035) were observed.

### 3.4. Ratings of Perceived Exertion (RPE)

RPE data for each set (SET1–SET4) and each experimental condition (CTR–SS1–SS2) are shown in Table 3. Statistical analysis revealed a significant effect of the SET factor on RPE (*p* < 0.001), but no differences were found between CONDITIONS (*p* = 0.496) or for the CONDITION × SET interaction (*p* = 0.178). Post hoc analyses showed progressive increases in RPE across sets, with significant differences between SET1 and SET3 (*p* < 0.001), as well as between SET1 and SET4 (*p* < 0.005). Significant increases were also observed between SET2 and SET4 (*p* < 0.05).

### 3.5. Blood Lactate Concentration [La]

**Figure 2:**
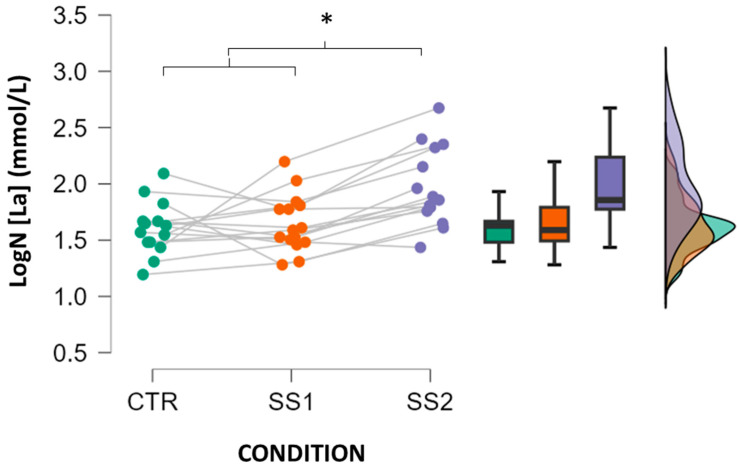
Blood lactate concentration measured 30 s before the final set (SET4) of the bench press exercise under each experimental condition (CTR, SS1, and SS2). * *p* < 0.05.

Individual [La] responses recorded under the different experimental conditions are shown in Figure 2. Statistical analysis revealed a significant CONDITION effect on lactate levels (*p* < 0.001). Post hoc analysis indicated that [La] values were significantly higher in SS2 compared to CTR (*p* < 0.001) and SS1 (*p* < 0.001). No significant differences were found between CTR and SS1 (*p* = 1.000).

### 3.6. Mechanical Performance During Antagonist Exercise (PBP)

For MSV, results revealed a significant main effect of the CONDITION factor (*p* = 0.011, η^2^_p_ = 0.401). No significant effects were found for the SET factor (*p* = 0.760, η^2^_p_ = 0.029) or the CONDITION × SET interaction (*p* = 0.332, η^2^_p_ = 0.083). No significant main effects or interactions were found for V_fastest_. MSV and V_fastest_ for PBP exercise are shown in Table 4.

## 4. Discussion

The present study provides evidence on the impact of different levels of fatigue induced by antagonist exercises in agonist–antagonist superset configurations. The main findings indicate that including antagonist supersets with low-to-moderate effort levels in the antagonist exercise does not compromise performance in the principal exercise and maintains similar RPE levels. Additionally, when the antagonist exercise is performed with a low effort level (4 out of 12 possible repetitions; SS1), [La] values are similar to those observed when performing the agonist exercise alone (CTR). On the other hand, when the antagonist effort is moderate (8 out of 12 repetitions; SS2), metabolic load (i.e., [La]) increases. Therefore, antagonist supersets with low-to-moderate effort appear to be an effective strategy to optimize training time by increasing volume in antagonist muscle groups without compromising performance in the main exercise. These findings have direct implications for strength coaches and practitioners. Agonist–antagonist supersets can enhance time efficiency while maintaining training intensity or acute mechanical performance, making them suitable for situations where training time is limited.

Although no significant differences were found in bench press lifting velocity between conditions (CTR, SS1, SS2), a progressive decrease in both mean and fastest velocity across sets was observed. This is in line with previous research reporting declines in mechanical performance in both superset and traditional strength training protocols [23,24]. Such performance loss reflects fatigue, potentially of both central and peripheral origin [21,25], which in this study was driven by accumulated volume in the primary exercise, not by the experimental configuration.

Contrary to our initial hypothesis, SS1 and SS2 conditions did not significantly affect bench press mechanical performance compared to the control condition. These results agree with findings from Weakley et al. [26], who noted that superset structure can influence some markers of fatigue but does not always compromise mechanical performance in the primary exercise. Furthermore, Robbins et al. [27] found that supersets do not reduce total volume when intensity and repetitions are controlled. One possible explanation is that fatigue induced by the antagonist exercise may not have fully transferred to the agonist muscles due to the distinct neural and mechanical demands of each movement [7]. Additionally, neuromuscular compensation mechanisms, such as increased motor unit recruitment or altered activation patterns, might have helped maintain performance in the agonist exercise [24]. Nevertheless, caution is warranted when interpreting these findings, since no direct mechanistic data were obtained to elucidate the physiological or neural processes underlying performance preservation in the superset condition.

In terms of RPE, a progressive increase was observed across sets in all conditions, with no statistically significant differences between them. However, other studies have reported significantly higher RPE under superset protocols versus traditional structures, attributed to shorter rest intervals and greater metabolic accumulation [9,28]. Thus, RPE behavior may depend on protocol structure (pairing type, effort level, rest time), as well as individual factors such as effort tolerance and training status. It is worth noting that in this study, RPE was assessed after each set of the agonist exercise (BP), unlike other studies that evaluated RPE post-session [9,26,28]. The session RPE (sRPE) method—assessed ~15 min post-session—captures total perceived effort and may affect how perceptual responses are interpreted.

Regarding metabolic response, lactate concentrations were significantly higher in the SS2 condition than in CTR and SS1. This aligns with Weakley et al. [9], who reported elevated lactate and creatine kinase levels after supersets and tri-sets. The meta-analysis by Zhang et al. [10] also found that supersets are associated with consistently higher lactate levels during and after exercise (SMD = 0.94 and 1.13, respectively), possibly reflecting increased energy demand. This increased lactate suggests greater anaerobic glycolysis, which not only raises internal load but may also promote metabolic adaptations with chronic application [9]. In fact, total energy expenditure after a superset session exceeds that of traditional structures, including increased post-exercise oxygen consumption, with potential applications in body composition programs [29,30].

The main limitations of the present study include the small and homogeneous sample (*n* = 14), which limits generalizability. The sample consisted solely of trained young men, preventing extrapolation to women, older adults, or untrained populations. Additionally, the study was acute in nature, so long-term neuromuscular or morphological adaptations, which are the primary goals of training, cannot be inferred. Furthermore, session RPE was not collected, which should be considered a methodological limitation, although set-by-set RPE assessment using the OMNI-RES scale provided valid and reliable perceptual data for the study purpose. Finally, the results could differ if antagonist sets are performed with greater levels of effort (e.g., repetitions to failure), but this strategy was not considered as it is not optimal to induce athletic performance gains [31].

## 5. Conclusions

Agonist–antagonist supersets represent an effective strategy to accumulate greater training volume for antagonistic muscle groups without increasing session duration, perceived exertion, or compromising acute mechanical performance in the principal exercise (i.e., the one given priority by being performed first). However, configurations involving greater volume in the antagonist exercise (e.g., SS2) may induce greater metabolic load, as indicated by increased [La]. Future studies should examine longitudinal adaptations when comparing traditional training formats (e.g., 4 sets of BP followed by 4 sets of PBP) with different agonist–antagonist superset configurations (e.g., SS1 and SS2 protocols).

## Figures and Tables

**Table 1:** Baseline wellness variables prior to training.

Variable	CTR	SS1	SS2	Χ^2^ (Friedman)	*p*
Sleep quality (a.u.)	7.13 ± 1.06	7.13 ± 1.25	6.47 ± 1.85	2.44	0.295
Fatigue (a.u.)	3.47 ± 1.25	3.73 ± 1.62	3.93 ± 1.91	2.33	0.312
Muscular pain (a.u.)	3.47 ± 2.36	3.13 ± 1.96	3.13 ± 1.77	0.81	0.668
Stress (a.u.)	3.07 ± 1.83	3.07 ± 2.05	3.20 ± 2.01	0.84	0.656

a.u. = arbitrary units.

**Table 2:** MSV and V_fastest_ during BP by condition and number of sets.

**Variable**	**Condition**	SET1	SET2	SET3	SET4
MSV (m·s^−1^)	CTR	0.549 ± 0.133	0.537 ± 0.127	0.527 ± 0.141 ^a^	0.510 ± 0.119 ^b^
	SS1	0.606 ± 0.148	0.548 ± 0.139	0.542 ± 0.137 ^a^	0.530 ± 0.131 ^b^
	SS2	0.564 ± 0.142	0.536 ± 0.145	0.529 ± 0.132 ^a^	0.520 ± 0.132 ^b^
V_fastest_ (m·s^−1^)	CTR	0.620 ± 0.156	0.614 ± 0.159	0.623 ± 0.170	0.594 ± 0.144
	SS1	0.648 ± 0.160	0.640 ± 0.163	0.644 ± 0.169	0.626 ± 0.145
	SS2	0.653 ± 0.179	0.633 ± 0.169	0.619 ± 0.144	0.611 ± 0.160

^a^ Statistically different from SET1; ^b^ Statistically different from SET1.

**Table 3:** RPE by condition and number of sets.

**Condition**	SET1	SET2	SET3	SET4
CTR ( a.u.)	5.79 ± 0.89	6.29 ± 1.07	6.57 ± 1.28 ^a^	6.57 ± 1.02 ^b,c^
SS1 ( a.u.)	5.93 ± 1.33	6.50 ± 1.16	6.64 ± 1.28 ^a^	7.00 ± 1.24 ^b,c^
SS2 ( a.u.)	5.79 ± 0.89	6.14 ± 1.17	6.64 ± 0.93 ^a^	7.14 ± 1.23 ^b,c^

a.u = arbitrary units; ^a^ Statistically different from SET1; ^b^ Statistically different from SET1; ^c^ Statistically different from SET2.

**Table 4:** MSV and V_fastest_ during the PBP exercise for each set (SET1–SET4) in the two antagonist superset sessions (SS1 and SS2).

**Variable**	**Condition**	SET 1	SET 2	SET 3	SET 4
MSV (m·s^−1^)	SS1	0.807 ± 0.174	0.816 ± 0.171	0.806 ± 0.159	0.820 ± 0.186
	SS2	0.764 ± 0.176 ^a^	0.757 ± 0.168 ^a^	0.761 ± 0.167 ^a^	0.734 ± 0.151 ^a^
V_fastest_ (m·s^−1^)	SS1	0.856 ± 0.184	0.865 ± 0.181	0.856 ± 0.163	0.868 ± 0.190
	SS2	0.849 ± 0.196	0.856 ± 0.181	0.873 ± 0.186	0.840 ± 0.152

^a^ Statistically different from SS1.

## Data Availability

Data may be requested from the corresponding author.

## References

[1] Ratamess N., Alvar B., Evetoch T., Housh T., Kibler W., Kraemer W.J., Triplett N.T., American College of Sports Medicine (2009). Progression models in resistance training for healthy adults. Med. Sci. Sports Exerc..

[2] Kraemer W.J., Ratamess N.A. (2004). Fundamentals of resistance training: Progression and exercise prescription. Med. Sci. Sports Exerc..

[3] Suchomel T.J., Nimphius S., Stone M.H. (2016). The importance of muscular strength in athletic performance. Sports Med..

[4] Schoenfeld B.J. (2010). The mechanisms of muscle hypertrophy and their application to resistance training. J. Strength Cond. Res..

[5] Schoenfeld B.J., Grgic J., Van Every D.W., Plotkin D.L. (2021). Loading recommendations for muscle strength, hypertrophy, and local endurance: A re-examination of the repetition continuum. Sports.

[6] Ekkekakis P., Parfitt G., Petruzzello S.J. (2011). The pleasure and displeasure people feel when they exercise at different intensities: Decennial update and progress towards a tripartite rationale for exercise intensity prescription. Sports Med..

[7] Robbins D.W., Young W.B., Behm D.G., Payne W.R. (2010). Agonist-antagonist paired set resistance training: A brief review. J. Strength Cond. Res..

[8] Paz G.A., de Freitas Maia M., Miranda H., de Castro J.B.P., Willardson J.M. (2020). Maximal strength performance, efficiency, and myoelectric responses with differing intra-set rest intervals during paired set training. J. Bodyw. Mov. Ther..

[9] Weakley J.J.S., Till K., Read D.B., Roe G.A.B., Darrall-Jones J., Phibbs P.J., Jones B. (2017). The effects of traditional, superset, and tri-set resistance training structures on perceived intensity and physiological responses. Eur. J. Appl. Physiol..

[10] Zhang X., Weakley J., Li H., Li Z., García-Ramos A. (2025). Superset Versus Traditional Resistance Training Prescriptions: A Systematic Review and Meta-Analysis Exploring Acute and Chronic Effects on Mechanical, Metabolic, and Perceptual Variables. Sports Med..

[11] Bentes C.M., Simão R., Bunker T., Rhea M.R., Miranda H., Gomes T.M., Novaes J.d.S. (2012). Acute effects of dropsets among different resistance training methods in upper body performance. J. Hum. Kinet..

[12] González-Badillo J.J., Sánchez-Medina L. (2010). Movement velocity as a measure of loading intensity in resistance training. Int. J. Sports Med..

[13] Weakley J., Mann B., Banyard H., McLaren S., Scott T., Garcia-Ramos A. (2021). Velocity-Based Training: From Theory to Application. Strength. Cond. J..

[14] González-Badillo J.J., Yañez-García J.M., Mora-Custodio R., Rodríguez-Rosell D. (2017). Velocity Loss as a Variable for Monitoring Resistance Exercise. Int. J. Sports Med..

[15] Flanagan E., Jovanović M. (2014). Researched Applications of Velocity Based Strength Training. J. Aust. Strength Cond..

[16] Robertson R.J., Goss F.L., Rutkowski J., Lenz B., Dixon C., Timmer J., Frazee K., Dube J., Andreacci J. (2003). Concurrent validation of the OMNI perceived exertion scale for resistance exercise. Med. Sci. Sports Exerc..

[17] Sánchez-Medina L., González-Badillo J.J., Pérez C.E., Pallarés J.G. (2014). Velocity- and power-load relationships of the bench pull vs. bench press exercises. Int. J. Sports Med..

[18] Janicijevic D., Jukic I., Weakley J., García-Ramos A. (2021). Bench Press 1-Repetition Maximum Estimation Through the Individualized Load–Velocity Relationship: Comparison of Different Regression Models and Minimal Velocity Thresholds. Int. J. Sports Physiol. Perform..

[19] García-Ramos A., Barboza-González P., Ulloa-Díaz D., Rodriguez-Perea A., Martinez-Garcia D., Guede-Rojas F., Hinojosa-Riveros H., Chirosa-Ríos L.J., Cuevas-Aburto J., Janicijevic D. (2019). Reliability and validity of different methods of estimating the one-repetition maximum during the free-weight prone bench pull exercise. J. Sports Sci..

[20] Hooper S.L., Mackinnon L.T., Howard A., Gordon R.D., Bachmann A.W. (1995). Markers for monitoring overtraining and recovery. Med. Sci. Sports Exerc..

[21] Márquez G., Romero-Arenas S., Marín-Pagán C., Vera-Ibañez A., Fernández Del Olmo M., Taube W. (2017). Peripheral and central fatigue after high intensity resistance circuit training. Muscle Nerve.

[22] Goodwin M.L., Harris J.E., Hernández A., Gladden L.B. (2007). Blood lactate measurements and analysis during exercise: A guide for clinicians. J. Diabetes Sci. Technol..

[23] Paz G.A., Maia M.F., Salerno V.P., Coburn J., Willardson J.M., Miranda H. (2019). Neuromuscular responses for resistance training sessions adopting traditional, superset, paired set and circuit methods. J. Sports Med. Phys. Fitness.

[24] Paz G.A., Robbins D.W., de Oliveira C.G., Bottaro M., Miranda H. (2017). Volume Load and Neuromuscular Fatigue During an Acute Bout of Agonist-Antagonist Paired-Set vs. Traditional-Set Training. J. Strength Cond. Res..

[25] González-Hernández J.M., García-Ramos A., Colomer-Poveda D., Tvarijonaviciute A., Cerón J., Jiménez-Reyes P., Márquez G. (2021). Resistance training to failure vs. not to failure: Acute and delayed markers of mechanical, neuromuscular, and biochemical fatigue. J. Strength Cond. Res..

[26] Weakley J.J.S., Till K., Read D.B., Phibbs P.J., Roe G., Darrall-Jones J., Jones B.L. (2020). The effects of superset configuration on kinetic, kinematic, and perceived exertion in the barbell bench press. J. Strength Cond. Res..

[27] Robbins D.W., Young W.B., Behm D.G. (2010). The effect of an upper-body agonist–antagonist resistance training protocol on volume load and efficiency. J. Strength Cond. Res..

[28] Corrêa Neto V.G., Silva D.D.N., Palma A., de Oliveira F., Vingren J.L., Marchetti P.H., da Silva Novaes J., Monteiro E.R. (2024). Comparison between traditional and alternated resistance exercises on blood pressure, acute neuromuscular responses, and rating of perceived exertion in recreationally resistance-trained men. J. Strength Cond. Res..

[29] Zhao H., Yamaguchi S., Okada J. (2020). Effects of rest interval array on training volume, perceived exertion, neuromuscular fatigue, and metabolic responses during agonist-antagonist muscle alternative training. J. Sports Med. Phys. Fitness.

[30] Kelleher A.R., Hackney K.J., Fairchild T.J., Keslacy S., Ploutz-Snyder L.L. (2010). The metabolic costs of reciprocal supersets vs. traditional resistance exercise in young recreationally active adults. J. Strength Cond. Res..

[31] Pareja-Blanco F., Rodríguez-Rosell D., Sánchez-Medina L., Sanchis-Moysi J., Dorado C., Mora-Custodio R., Yáñez-García J.M., Morales-Alamo D., Pérez-Suárez I., Calbet J.A.L. (2017). Effects of velocity loss during resistance training on athletic performance, strength gains and muscle adaptations. Scand. J. Med. Sci. Sports.

